# Incorporation of Socio-Economic Features' Ranking in Multicriteria Analysis Based on Ecosystem Services for Marine Protected Area Planning

**DOI:** 10.1371/journal.pone.0154473

**Published:** 2016-05-16

**Authors:** Michelle E. Portman, Ateret Shabtay-Yanai, Asaf Zanzuri

**Affiliations:** 1 Faculty of Architecture and Town Planning, Technion – Israel Institute of Technology, Haifa, Israel; 2 The Porter School of Environmental Studies, Tel Aviv University, Tel Aviv, Israel; University of Missouri, UNITED STATES

## Abstract

Developed decades ago for spatial choice problems related to zoning in the urban planning field, multicriteria analysis (MCA) has more recently been applied to environmental conflicts and presented in several documented cases for the creation of protected area management plans. Its application is considered here for the development of zoning as part of a proposed marine protected area management plan. The case study incorporates specially-explicit conservation features while considering stakeholder preferences, expert opinion and characteristics of data quality. It involves the weighting of criteria using a modified analytical hierarchy process. Experts ranked physical attributes which include socio-economically valued physical features. The parameters used for the ranking of (physical) attributes important for socio-economic reasons are derived from the field of ecosystem services assessment. Inclusion of these feature values results in protection that emphasizes those areas closest to shore, most likely because of accessibility and familiarity parameters and because of data biases. Therefore, other spatial conservation prioritization methods should be considered to supplement the MCA and efforts should be made to improve data about ecosystem service values farther from shore. Otherwise, the MCA method allows incorporation of expert and stakeholder preferences and ecosystem services values while maintaining the advantages of simplicity and clarity.

## Introduction

Of note among new conservation initiatives announced at the International Union for the Conservation of Nature's decadal World Parks Congress in 2014 was the emphasis on commitments to protect the *marine* environment. A document, developed by congress participants and named "The Promise of Sydney", solidifies efforts to protect 10% of the world’s oceans by 2020, mostly by the establishment of marine protected areas (MPAs). However, establishment may not be enough. MPAs require good management plans that protect habitat and marine organisms from threats caused by human uses occurring within them [1]. Research has shown that the consideration of human dimensions, particularly socio-economic interests of user populations, is important for the development of management plans (e.g., [2, 3]); such interests can be integrated using parameters taken from the field of ecosystem service assessment.

MPA management plans often designate place- and depth-based "zones". Zones facilitate understanding and encourage compliance by those who have a stake in management and use of a marine area [4–6]. "Zoning" is defined as a "set of regulatory measures" used to implement spatial planning. Specifically, a zone accomodates certain uses, or different levels of use. Regulations for the zones address prohibitions and/or permitted uses [7] and are usually accompanied by conditions for use or protection such as complete or temporary closures, equipment constraints, permits, and economic incentives/disincentives [1, 8, 9]. But what is the best way to arrive at the configuration of these zones?

Multicriteria analysis (MCA) emerged several decades ago as a decision-making tool for designing spatially-explicit zoning in the field of urban planning [10, 11]. Since then it has been used extensively in the environmental field [12–15], for conservation (e.g., [16–18]), and for protected area planning and management (e.g., [19, 20]). Its advantages include the ability to integrate various types of data (i.e., qualitative and quantitative) and to give expression to stakeholder priorities (e.g., [17, 21, 22]).

The weighting and scoring of criteria usually incorporates preferences or opinions arrived at through surveys that employ methods such as analytic hierarchy process (AHP), multi-attribute utility theory, and outranking [13]. Cases in point include a study of the Asinara Marine Reserve [21] and the proposed Red Sea Marine Peace Park [23]. However, this scoring is often divorced from the spatial layout of important features within areas of interest and attribute data quality. Although MCA was used to arrive at zoning for the above mentioned MPAs, quality characteristics of the data were not incorporated (e.g., measures of certainty/uncertainty); physical attribute scores were applied largely in blanket fashion for criteria “layers” and without consideration of ecosystem service values.

This research proposes opportunities for scoring within *a single category* of attribute, thus providing further opportunities to combine both expert opinion and stakeholder preferences and using criteria related to ecosystem services (ES) values. In addition to the scoring *between* layers of information through the administration of surveys, weights are applied to features based on: 1) spatial characteristics (i.e., size and location), and 2) relative contribution. A feature's relative contribution considers data quality concerns, such as certainty and confidence in the data.

The use of this method is demonstrated through the development of proposed zoning for the expansion of an existing MPA off the coast of Israel. The proposed Rosh Hanikra MPA offers a plethora of seascape features (i.e., underwater ridges, chasms, and canyons) and thus is an exemplar of the Eastern Mediterranean's unique marine and coastal ecosystems and one for which MCA can make a significant contribution. Also, relative to other areas along the Israeli coastline, data is available for a number of criteria that can be used for the analysis.

### Planning’s Contribution to MPA zoning

Urban planning and spatial conservation prioritization share an interest in the treatment of choice problems [11, 24]. For the most part, the solving of spatial choice problems in the planning field has developed in parallel to the use of spatial conservation prioritization. Although many case studies of such systematic conservation planning exist [2, 25–29], studies of decision-making methods adopted from the planning field that combine both socio-economically important features (particularly those using the ecosystem service approach) and features important for conservation are in short supply.

Planning practitioners use several methods for the evaluation of alternatives or choices (initially referred to as “situations”) by means of a number of multidimensional evaluation criteria. While there is criticism of the use of MCA, and "scoring" methods in general for spatial conservation prioritization, including many questions related to their use (e.g., [20, 24]), research on their potential for such purposes is limited. In Huang et al.’s [13] review of 312 papers presenting cases of MCA being used for environmental decision-making, protected area management is not even designated as a category of application.

The planning of protected area zoning—frequently used for protected area management—requires choosing among possible scenarios. Therefore, MCA is highly applicable despite overall goals and physical attributes of concern being distinct from those considered for zoning neighborhoods and cities. The method's applicability hinges on the fact that conservation planning, like all planning, calls for stakeholder input and should consider people as part of the ecosystem of concern [17, 22, 30, 31]. MCA provides opportunities for such input [13].

Perhaps even more so than for terrestrial protected areas, due to the public nature of the marine environment, the design of MPA zones should incorporate involvement of local community members and resources users [22, 32, 33]. Indeed, if we look beyond the use of MCA for protected area planning, we find several uses of MCA in the marine resource management literature, particularly for tourism and recreation [19], for sustainable fisheries management [34–36] and recently for marine spatial planning [37]. In some cases, MCA has been used without a spatial component [19], meaning without giving *spatial* expression to the outcomes of the decisions made (as would be the case with the provision of a zoning proposal).

Much of the emphasis in MCA literature aimed at resource conservation focuses on the soliciting of stakeholder (resource users' and experts') preferences for criteria weighting (e.g., [17]). Methods commonly used for the weighting of multiple criteria include weighted summation, ideal/reference point and outranking methods [38]. The pairwise method, AHP, has been used frequently for MCA related to conservation [13, 16, 38]. In their review of the use of MCA specifically tailored to conservation area networks, both Moffett & Sarkar [27] and Stager & Rosenberger [17] recommend the use of AHP for arriving at stakeholder weights.

The importance of addressing the spatial component of MCA for resource conservation contrasts with its extensive treatment in the *general* MCA literature within which spatial layout is often not of concern (see [38]). This study posits that the spatial component of analysis is particularly critical when using MCA for conservation planning in view of the documented challenges to inclusion of ecosystem service values in decision making [32, 39]. Ecosystem service values related to locational characteristics can be readily used as criteria. Such an approach broadens the MCA such that socio-economic criteria are incorporated, together with criteria supporting nature conservation.

### Shortcomings of Multicriteria Analysis

A number of review papers discuss and analyze MCA approaches in the context of environmental work, albeit from different angles. Malczewski [32] focuses on GIS-based MCA results in the analysis of a number of conservation applications, among other general applications. Huang et al. [13] covers a broad range of environmental applications, but few cases in this review involve the spatial layout of features or a concern for biodiversity or ecosystem health. Most of the papers reviewed by Huang et al. [13] focus on how MCA can aid decision-making through trade-offs between preferences. The most relevant of the reviews to this research are Moffet & Sarkar [27] and a fourth case study comparison of the use of MCA for conservation conflicts by Davies et al. [18]. Moffet & Sarkar's [27] paper, termed a “mini-review” by the authors, deals exclusively with the use of MCA for the design of conservation area networks.

**Fig 1:**
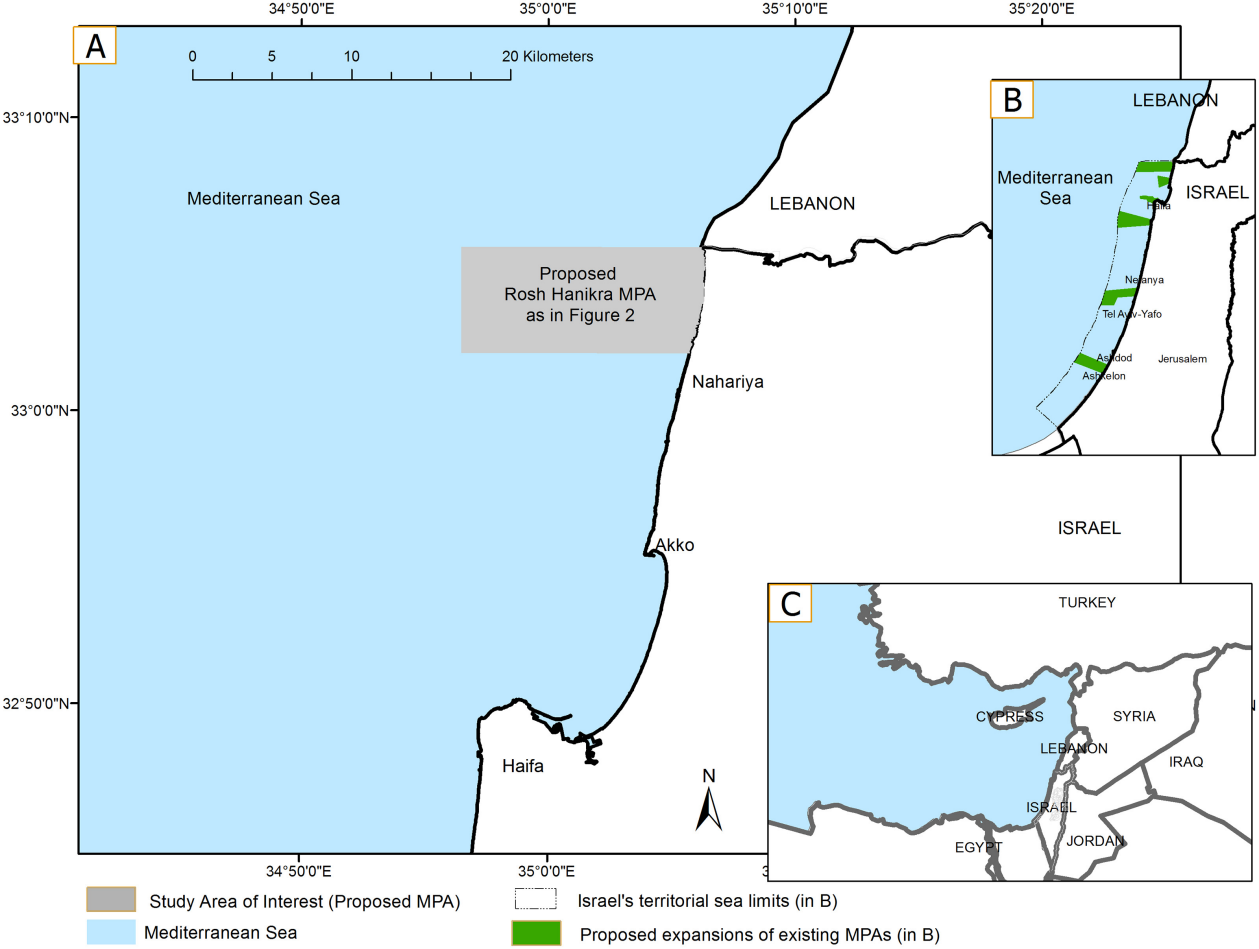
The area of interest (AOI): the proposed MPA. (A) Detailed view of the currently proposed Rosh Hanikra protected area. (B) The Mediterranean Sea coast of Israel with the six new and/or expanded reserves proposed by the INPA (in green). The northernmost proposed MPA is the area originally proposed for expansion by the INPA. (C) Locus map of Israel among other countries of the Eastern Mediterranean.

Moffett & Sarkar [27] suggest that MCA is inappropriate for decision making about conservation area networks when there is uncertainty (see Fig 1 in Moffet & Sarker [27]). Ferrier & Wintle [40] critique MCA methods based on scoring of desired species or other conservation features on the grounds that the top-ranked sites frequently contain similar sets of species while missing others. Other MCA literature describes the ranking (or scoring) used in MCA as simple to carry out but often lacking in ability to express real world complexities [41].

In the conservation context, MCA usually results in the scoring of areas as suitable for different levels of protection [21, 23]. Despite its importance to the calculation of concordance scores (on par with the stakeholder preferences or “weights”), the ranking of physical attributes has rarely been the emphasis of applied research. "Physical attributes" refer to those elements of the environment that make up an area of interest's significant features. Thus, ranking will indicate the conditions and value of features; ranks will be high for quality attributes (e.g., unique or endangered habitat) and low for less important attributes.

A notable exception for environmental sensitivity studies is Svoray et al. [16] for which the ranking of physical attributes using a habitat heterogeneity model is central to its contribution. However, Svoray et al.'s model was developed for the identification of ecological sensitive areas in an urban planning context and not protected area planning. Also, the extent of information available for terrestrial conservation was far beyond that commonly available for decision making about in the marine environment [1, 42, 43] and the study failed to consider in any way the provision of ecosystem services or the certainty of physical data.

Some researchers have acknowledged the role of uncertainty in general MCA decision making processes. Of the 319 studies reviewed by Malczewski [38], 23% were considered to be made under conditions of uncertainty, rendering decision making stochastic or "fuzzy". With no connection to MCA, conservation planners have highlighted the importance of moving forward even when data is uncertain or incomplete [44]. Sometimes the use of incomplete data for MCA is encouraged [18]. Rejecting such data could lead to the exclusion of legitimate social values, undermine the fairness of the process and possibly increase the risk that any resulting decisions may not achieve the desired environmental or socio-economic outcomes. This study posits that the problem is not so much the uncertainty or incompleteness in itself, but the failure to acknowledge these aspects in the scoring of environmental attributes.

### The Rosh Hanikra Case Study

The Mediterranean basin has been particularly impacted by development, land-based marine pollution, invasive species, habitat loss and overfishing [31, 45–48]. To address and ameliorate the situation, two important regional agreements, the Convention on Biological Diversity and the Convention for the Protection of the Mediterranean Sea Against Pollution call for the establishment of MPAs. Despite interest in meeting these goals as a member of these conventions, Israel has very limited "marine reserves" to date; the existing reserves extend at most only several hundred meters from the shore. Recognizing the need to be proactive, the Israel Nature Parks Authority (INPA), recently proposed six new (mostly expanded) marine reserves that cover a relatively significant portion of Israel's territorial waters in the Mediterranean Sea [49] (Fig 1B).

The Rosh Hanikra MPA (hereafter "Rosh Hanikra") is the first of these expansions to be considered for approval. Israel’s planning law requires a plan detailing the expansion to be approved for public review prior to final approval by the country's Northern District Planning Commission. Thus it is critical to stipulate in the MPA management plan what protections are proposed in which areas because citizen and planning officials will likely file objections and comment on the plan based on these restrictions and prohibitions. A zoning plan for the MPA could help in this regard [46].

The expanded Rosh Hanikra MPA is slated to cover approximately 100 km^2^; it will stretch along 5 km of the northernmost part of the 190 km-long Mediterranean shore of Israel and extendwestward from the shore for approximately 15 km (Fig 1A). This proposal has advanced before others due largely to its peripheral location and outstanding qualities including an underwater canyon and islets that provide important habitat. It has coastal scenic value—a small range of soft limestone hills slopes down to the water where dramatic grottos descend into shallow lagoons [50].

Rosh Hanikra's submerged area is rich with a variety of fish and Gastropoda, with more than 60 species documented. Such variety in such a small area has been found only in the Red Sea. The Loggerhead Sea and Green Turtle (*Caretta caretta* and *Chelonia mydas* respectively) have hatching sites along the beach [50] and the endangered Mediterranean Monk Seal (*Monachus-Monuchos*) was recently sited within the proposed MPA’s boundaries after being absent for many years [51]. The imprint of ancient cultures and local heritage is also significant in the Rosh Hanikra area, with elaborate archeological sites drawing many visitors to the coast. Within the area proposed for the expanded reserve, the remains of the Achziv harbor are testament of the important port that existed here from the Bronze Age until the time of the Crusaders [50].

## Methods

The MCA involves the use of two main sets of numerical values. The first set consists of those values that indicate preferences for each of the four (high-level) criteria, each in relation to the other three; the second set are those values that indicate the quality of physical attributes, the “criteria”. High-level criteria, as explained in detail below, are: seascape values, water sports values, commercial values and natural marine values. Each higher-level criteria group is composed of a number of lower-level criteria as in other MCAs [16, 19, 21]. The area of analysis, its boundaries and thus its physical characteristics have been previously determined by work carried out in the past by the INPA. This analysis seeks to develop proposed zoning (given that the area within the boundaries is already proposed as an MPA), based on which areas are best suited to protection of various levels.

These two sets of data led to the development of "concordance scores". First, the study used a modified AHP to determine user preference weights (as in [17, 27]) from among the higher level-criteria (as in [21, 23]). In parallel, experts valued feature attributes based on the quality of the information, particularly certainty and completeness, and otherwise used an ES approach. In the last stage, a widely-used MCA algorithm (see [21]) was applied to calculate the "concordance scores", thus indicating suitability to three protection levels, termed “scenarios”.

The following sub-sections describe how the two main sets of numerical values were arrived at under the three scenarios followed by a description of how the concordance scores are calculated. The emphasis in this section is on the method used to arrive at the rankings of the physical attributes, i.e., elements of the environment as understood based on the "services" they provide to stakeholders and users of the area.

### Stakeholder weights

Stakeholders ranked the higher level attributes under three protection scenarios: fully protected (FP), seascape reserve (SR), and marine park (MP) using questionnaires administered in the field. (The Technion Social and Behavioral Sciences Institutional Review Board approved the surveying done.) Each scenario led to a different spatially explicit zoning proposal. The scenarios are based on the protection levels provided in Israel's National Outline Scheme for Nature Reserves and National Parks in order of decreasing stringency: nature reserves, landscape reserves and national parks.

For this study, the most rigorous protection scenario (FP), aims to conserve biological and genetic resources representing the ecosystem(s) of the reserve. Under the FP scenario, no entry will be allowed by potential users other than for research purposes (see a list of user groups in Table 1) in much of the MPA (the AOI in Fig 1). Management under the mid-level protection scenario (SR), aims to achieve sustainable use of marine resources. Activities will be minimal and controlled with entry and use allowed only in certain areas; conditions and prohibitions will be use-specific and spatially explicit. Under the least restrictive scenario (MP), management focuses on human use while protecting unique natural features found within reserve boundaries. Under this scenario most of the MPA will not have any entry and use restrictions whatsoever.

**Table 1:** Stakeholder (rounded) weights derived from the eigenvectors of the pairwise comparison questionnaire.

	Fully Protected (FP)				Seascape Reserve (SR)				Marine Park (MP)			
High-level criteria	SV	WS	CV	NMV	SV	WS	CV	NMV	SV	WS	CV	NMV
Academic Experts(8)	1.613	0.454	0.291	4.209	1.630	0.590	0.319	3.977	1.908	1.695	0.646	2.927
Scuba divers (7)	1.059	1.165	0.346	4.176	1.792	1.924	0.734	2.925	1.428	2.584	0.730	2.549
Fisherman (8)	2.232	2.983	1.030	1.533	1.570	2.603	0.810	1.849	1.345	2.357	0.857	2.192
Recreationists (7)	2.295	1.891	0.401	3.770	2.170	1.960	0.535	3.008	2.034	2.289	0.545	3.194
Tourists (10)	2.169	0.690	0.700	3.416	2.020	1.167	1.034	2.627	1.566	2.547	1.642	1.942
Reserve employees (7)	1.770	0.897	0.730	4.037	1.595	1.226	0.726	3.814	1.961	1.725	0.848	3.193
Field experts (12)*	1.601	0.661	0.355	4.437	1.938	1.035	0.500	3.552	1.871	1.881	0.738	2.659
**Total average (59)**	**1.820**	**1.249**	**0.550**	**3.654**	**1.816**	**1.501**	**0.666**	**3.108**	**1.730**	**2.154**	**0.858**	**2.665**

Under each scenario, the four high-level criteria were valued by respondents in relation to each other: seascape values (SV), Water sports values (WS), commercial values (CV), and natural marine values (NMV).

*Marine biologists; most working in the field.

The above three protection level scenarios were described in the administered questionnaires; survey administrators working in the field asked respondents to rank the four higher-level criteria. A total of eighteen questions were presented to the stakeholders in recurring sets of six questions, one set for each of the three scenarios. As in a typical AHP process, stakeholders indicated preference for each of the four high-level criteria (explained in detail in the next section) by comparing them in pairwise fashion (See S1 Appendix).

The questionnaires were distributed on-site in paper form in Arabic, English or Hebrew within and around the existing Rosh Hanikra reserve, while targeting commercial and visiting areas. Sites for administering the questionnaire depended to some extent on the user group. For example, fishermen who work away from tourist areas were targeted along the beach and some marine biology professionals were approached in their offices. Participants provided *only* verbal consent (as required by the researchers' Institutional Review Board) in order to avoid identification. Verbal consent is registered as having been given by the written response in each survey document.

The number of choices available to the respondent were limited to five for convenience and conciseness. This is in line with Saaty [52], credited with developing the pairwise comparison method, who determined that to avoid confusion the number of choices should be no higher than seven (± 2). Questionnaire responses provided the averaged weights extracted from each matrices’ associated eigenvalue. These averaged weights ***w***, from among 0 <***w*** <1, indicate the relative importance the 59 survey respondents gave to each of the four criteria groups ***k***. Resulting preference weights (Table 1) give an indication of how criteria are valued under changing scenarios by constituent groups. Total averaged scores (of all groups; see Table 1) along with the physical attribute data, were used for calculation of the concordance scores.

Survey respondents provide information on their preferences without relating these to spatial location, i.e., without any connection to cell location. Therefore, survey respondents do not know how their answers in the questionnaire will influence zoning until the final maps are produced. Advantages to such an approach are twofold. First, survey respondents avoid expressing support for any particular spatial plan. Secondly, modifications in the physical attribute data can be made independently (once new information on the physical environment becomes available or is updated and added to) without having to re-administer the survey [23].

### Physical attributes of the marine and coastal environment

Physical attribute data were ranked according to biotic and abiotic elements within the area of interest (AOI) (based mostly on secondary sources) and socio-economic attributes. In keeping with seminal ecosystem services literature (Table 2), socio-economic attributes include those valued for their aesthetics, commercial (e.g., higher rankings for featured areas used by fishermen and for water sports) and other use values.

**Table 2:** Explanation and sources for the choice of parameters for each high-level criteria.

Parameters	Explanation	Data analysis/collection method	Seminal Sources
Visibility (SV)	View capability from features without special gear	Buffers and opinion	[53–55]
Contribution to seascape (SV)	Contribution of feature to the unique (visual) seascape experience. Ex: the islets attract sea-birds that add to the user's "beach" vistas	Expert opinion	[56, 57]
Distance from shore (SV)	Inverse distance: the greater the distance, the lower the grade	Measurement (GIS)^a^	[53]
Use density (WS)	Percent of feature’s users from among all users in the AOI relative to the size of the feature. Higher values indicate higher use density. Ex: high percentage indicates a large number of visitors in a small feature area	Observation (surveying) and measurement (GIS)	[55]
Accessibility (WS)	Public accessibility (without special gear). Buffers around the features are respectively: nearby ≤ 50; mid-distant ≥ 50 and ≤100; distant: ≥ 100. Higher grades indicate proximity	Measured (GIS) buffers	[54, 55]
Cultural importance (WS)	Archeology and recreational fishing of highest value. Other (lesser) values: bathing beaches (mid-values), kayaking, surfing and diving (lowest value)	Expert opinion	[54, 55]
Social importance (WS)	Public and non-material component of well-being. In descending order: archeology and nearby bathing beaches, distant bathing beaches; other recreational uses, including fishing	Expert opinion	[56, 58]
Accessibility (CV)	The same as public accessibility for WS (above)	Measured (GIS) buffers	[54, 55]
Cost (CV)	Cost indicates a willingness-to-pay such that distant features used commercially will have a higher value	Measurement (GIS)	[54]
Seasonality (CV)	Lower grades for uses limited to weekends/holidays and certain seasons; higher grades for year-round uses (i.e., recreational fishing)	Expert opinion	[55]
Number of species (NMV)	Number of species relative to feature area	Raw data analysis or secondary source reports	[46, 59, 60]
Habitat uniqueness (NMV)	Uniqueness and sensitivity of habitat based on hard and soft seabed surface	Secondary source report	[49]
Certainty/Accuracy (NMV)	Accuracy of data according to source. Ex: direct measurement of fish and invertebrate species around the islets (i.e., [59]) results in higher scores than features scored using secondary source data (i.e., [46])	Raw data analysis or secondary source reports	[46, 59, 60]

^a^ Indicates the use of geographic information system (GIS) application

The numerous physical attributes (low-level criteria) were organized into the four “high-level" criteria: seascape values (SV); water sports/cultural values (WS); commercial values (CV); and natural marine values (NMV). The first category, SV, refers to seascape features such as: abrasion tables, submarine canyons, and inaccessible archaeological sites. WS values are attributes of cultural importance and those areas that support active and passive recreational activities such as for diving, surfing, snorkeling and archaeological exploration. For this group of values, sports are conducted on an individual basis, i.e., without the support of a place-based organized business. CV refers to areas used commercially for fishing, boat tours (tourism) and sports which are distinguished from the WS criteria by being dependent on organized business (including inaccessible archeological sites). Lastly, NMV refers to the quality of ecosystem-related biotic and abiotic resources such as species composition and geological features that provide important habitat.

The grades given to the criteria (referred to hereafter as "grading") consider different aspects of the spatial array as dependent on high-level criteria *type* (Table 3). Overall, grades depend on three main parameters: 1) spatial location of the feature; 2) contribution to socio-economic well-being; and 3) relative contribution within each high-level criterion. In regards to location, the high-use foci are for the most part based on distance from shore. For example, for seascape values, aesthetics is a major consideration; because visitors and resources users onshore value views, distance from the beach is a parameter influencing the grade.

**Table 3:** The features (column 2) making up each of the four high-level criteria (column 1).

(1)	(2)	(3)	(4)
High-level Criteria	Features	Ranking parameters	Grading convention
	(+spheres of influence)	(% of total high-level criteria value)	(normalized)
Seascape values (SV)	Submarine canyons	Visibility (50%)	1–5 (1 = lowest)
	Abrasion tables		
	Visible archeological sites	Contribution to seascape (15%)	1 = low; 5 = high
	Seaview: <4.7 km from the shore	Distance from shore (35%)	Inverse distance in meters^a^
	Islets		
Watersports/Cultural values (CV)	Accessible archeological sites	User density (20%)	0 ≤ 1
	Inaccessible archeological sites	Accessibility (50%)	1–5 (l = lowest)
	Beaches (nearby, mid-distant, distant)	Cultural importance ^b^ (15%)	1–5 (1 = lowest)
	Sites for kayaking, recreational fishing, surfing	Social importance^c^ (15%)	1–5 (3 = lowest)
	Entire area of interest		
Commercial values (CV)	Sites for diving, kaying, recreational fishing and other (organized) tourist activities	Accessibility (33%)	1–5 (3 = lowest)
		Cost (33%)	Distance from shore
		Seasonality (33%)	1–5 (2 = lowest)
Natural Marine Values (NMV)	Entire area of interest	Number of species (50%)	0–1
	Islets (100%)^d^	Habitat uniqueness (25%)	0–5
	Islets: 400 m buffer (75%)^d^	Certainty /Accuracy (25%)	0–1
	Islets: 401–1000 m buffer (50%)^d^		
	Deep sea		
	Continental slope and canyons		
	Continental shelf		
	Big canyons		
	Kurkar ridges		
	Continental ridges slope		
	Kurkar rocks near shore		

Expert opinion contributions are in columns 2 and 3.

^a^relative to the seaward extent of the valued feature farthest from shore (i.e., the submarine canyons).

^b^ Cultural use potential: archeology (all) and recreational fishing = 5; nearby bathing beach = 4; distant bathing beach = 3; kayaking, surfing and diving = 1.

^c^Social use potential: archeology (all) and nearby bathing beach = 5; distant bathing beach = 4; diving, kayaking, recreational fishing = 3.

^d^Reduced weight of data in buffers by distance.

The sub- (low-level) criteria were graded using parameters appropriate for each high-level criterion (SV, WS, CV and NMV). For example, for the SV features (explained below) we use three parameters: visibility, contribution to the seascape, and distance from shore. Each of these parameters was ranked for importance by experts (with a background in marine biology and familiarity with the AOI). These parameters are respectively 50%, 15%, and 35% of the final high level criteria grade. The sub-criteria grades were normalized before being summed to arrive at the high-level criteria grade (*e*_*ijk*_ in (Eq 1) below). Similarly, for the NMV, grades reflect certainty about the data collection; sub-criteria rank lower when farther from known data points.

#### Features

The most important (and unique) aspect of the method's physical attributes' grading system, is that of "features". Features consist of unique characteristics expected to make a significant contribution to either socio-economic or conservation purposes of the MPA. For example, for the SV category, features are submarine canyons, abrasion tables, sea views and islets. The parameters allow the grading of these features.

The grading parameters are derived from feature qualities. Most grades capture ecosystem service values, the widely-accepted currency used for judging ecosystem attribute benefit and worth [39, 61]. Due to their importance, each parameter is considered under its respective high-level criteria (see Table 3). Parameter units are given ranks (from one to five, with five being of greatest value) but some incorporate physical measures, such as distance. All parameters were normalized to account for unit differences. The ranks and measures were summed to arrive at the final score, *e*_*ijk*_ (0–4) where 0 indicates the least suitable for protection under the scenario and 4 the most suitable.

### Final Concordance Scores

Concordance scores are the results of (Eq 1): the value of all the sign functions of *e*_*ij*_ multiplied by the weights and summed for the for value *c*_*ijk*_ where *k* represents the scenarios: FP, SR and MP.

$$C_{ij}=\sum_{k}W_{k}\sum_{i\prime j\prime }\text{sgn}(e_{ijk}-e_{i\prime j\prime k})$$ (1)

Where

$$sgn\left(e_{ij-}e_{i^{\prime }j^{\prime }}\right)=[-1\;if\;e_{il}<e_{i^{\prime }j^{\prime }};0\;if\;e_{il}=e_{i^{\prime }j^{\prime }};1\;if\;e_{il}>e_{i^{\prime }j^{\prime }}$$ (2)

A grid "cell" refers to the p raster representation of the region partitioned into a systematic set of analysis areas (in this case 25 m^2^ cells) covering the AOI (*n* = 161,163). In the first step, the algorithm compares every cell (denoted by *e*) to every other cell in the spatial representation (hereafter: "grid") using the sign function portion of (1) which serves to consolidate the value of the cell by relating it to that of all other cells.

**Fig 2:**
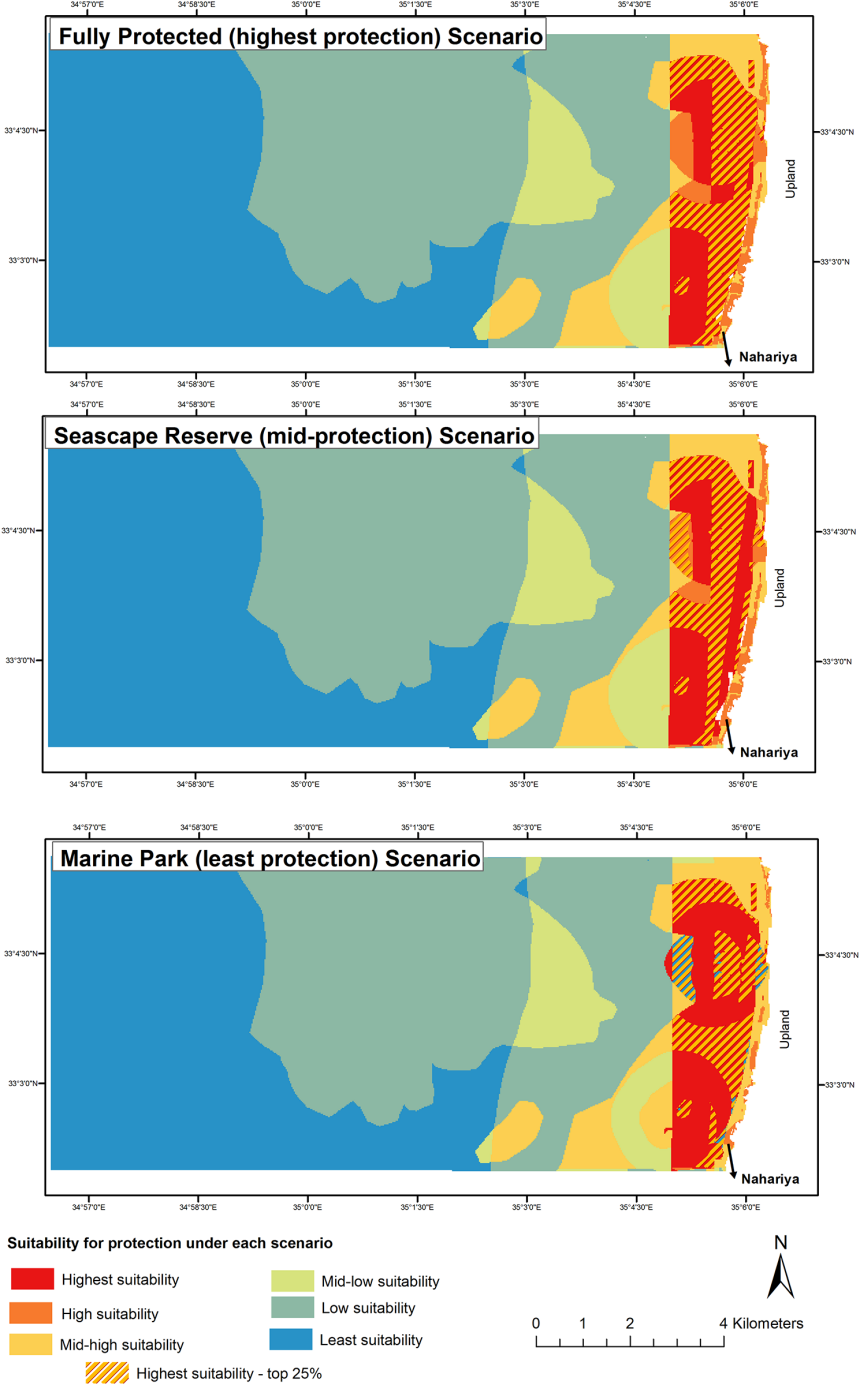
MCA results under different scenarios within the area of interest (as in Fig 1). From most restrictive to least: (A) fully-protected. (B) seascape reserve. (C) marine park. A significantly larger area of the AOI is indicated as suitable for protection under scenario (A) when considering the top quartile scores (≥ 25%).

The best way to compare each cell to all the other cells is through the use of a matrix with two axes: *i* and *j*. For example, the first cell *e*_*i1*_ is compared in turn to cells *e*_*i2*_, *e*_*i2*_, *e*_*i3*_…..(where *i* = 1 is the first row of the matrix) and so on until it has been compared to all the other cells of that higher-level criteria coverage (grid) layer. Values resulting from each comparison accumulate to cell *e*_*i1*_ so that its value indicates its relation (i.e., relative superiority or inferiority) to all other cells in the grid (see S2 Appendix). The second step consists of multiplying the resulting cell values by the stakeholder weights ***w***_***k***_ determined under each zoning scenario, thus resulting in ***C***_***ij***_ as mapped in Fig 2.

## Results

Areas of sensitive “valued” features show up as areas most suitable for high levels of protection. The method allowed comparison between the three scenarios. The FP scenario shows the most "nuanced" variation of suitability; the MP scenario shows significantly less features with high suitability. Given the small differences between the three outcome maps at the 25% upper quartile threshold, the FP scenario provides a reasonable starting point for determining boundaries of a no-take zone.

The threshold (shown in Fig 2) of the upper 25% (quartile) of the scores indicates protecting 5.275 km for the fully protected scenario, 4.835 km for the seascape reserve and 4.6 km for the least protected scenario. Further planning review (such as that conducted by the Northern District Planning Commission) could establish a different threshold value and thus produce map outcomes accordingly to arrive at exact zone boundaries.

Since the feature area and expert opinion scores remain constant between the scenarios with differences driven only by stakeholder preferences, we see greater complexity (more areas of high protection suitability) when users are asked to consider a greater protection scenario. For all scenarios, those areas closest to shore and around the islets, are most valued. Only near shore areas were those indicated even when the top 50 percent threshold was used for all three scenarios.

Results of the analysis in each of the scenarios show that areas considered as most suitable for protection are those close to shore. This important finding has repercussions for the use of ecosystem service values in MCA for the design of protected area zoning. It suggests that MCA of this type, that incorporates socio-economic criteria based on ecosystem services values tends to favor areas with higher usability while neglecting those areas with low user activity and accessibility.

When interpreting the results shown in Fig 2 it is important to note that multiple types of ES (such as sea views) have been considered. This renders other traditional "protected area" targets on par with socio-economic concerns represented by the physical attributes that make up the high-level criteria, such as those related to commercial values and those valued for water sports. If the areas indicated by the yellow markings (Fig 2) are those most suited for protection in each scenario, decision making can be "narrowed" to choosing between these alternatives. If policy makers can be persuaded to adopt the fully-protected scenario, it would likely provide the greatest area of protection as compared from among the areas indicated using the upper 25% threshold.

## Discussion

Although the conservation of the natural marine environment should be considered the fundamental objective of MPAs, research tells us that neglecting the socio-economic values in developing MPA management plans impedes their success [22, 62]. By incorporating stakeholder values, through preferences scoring and using ecosystem service valuation through the grading of attributes expressed spatially in various units (monetary, physical, qualitative, etc.), MPA management is improved.

The use of stakeholder weights to indicate trade-offs acceptable to the community of users is one of MCA's greatest advantages (e.g., [22, 23, 27]) and it allows the achievement of high levels of protection through the designation of the restrictions to specific feature areas. However, a couple of issues need to be pointed out. First, as mentioned, use of a method that gives significant weight to ecosystem services of different types (cultural, socio-economic, etc.) will likely recommend higher protection for areas that are close to shore. The results (Fig 2) show that features closer to shore ended up (by and large) with higher concordance scores, indicating higher suitability for greater protection, due to the importance of accessibility and other parameters associated with certain ecosystem services. For example, for users of the area to benefit from the recreational value of water sports, the area needs to be accessible. Such results are inextricably linked to the physical attribute data that were scored based on a combination of expert opinion about parameters mentioned in the literature as indicated in Table 2.

A second point relates to priorities of conservation in relation to priorities of ES. Other studies that have addressed protected area design (e.g., [24, 26]) and particularly those addressing the design of networks of protected areas [2, 25, 46] emphasize spatial association and connectivity in their analyses. While a method that considers spatial association to achieve conservation goals, such as persistence of species (as in [26, 63]), could be used to determine suitability of cells for protection, in the Rosh Hanikra case, the method applied renders spatial association for achievement of conservation goals less important. The reasons for this are threefold.

First, boundaries of the MPA have already been determined; all areas of the AOI are destined to be included, so a binary situation of inclusion or exclusion does not exist as it may in other cases. The issue at hand is the *level of protection* to be delineated by zoning. Secondly, attribute values address a variety of ES, among which nature conservation (i.e., supporting services) including species persistence or biodiversity, would be only one of the high-level criteria. Important values in this study include other parameters related to ES, e.g., aesthetics and access. For most of these values, spatial association (dispersal or concentration of the most highly protected areas) are not factors. Thirdly, there is a sampling bias in the natural marine values (NMV) data such that spatial association for chosen species is not clearly discernable from the available data. The data used in this study does not necessarily reflect those species most valued or most endangered, although the sampling bias is dealt with by the use of buffers and weighting (by experts) of the physical attribute data such that attributes father from sampling stations have less influence on the overall high-level criteria grade (see Table 3 under NMV).

The use of criteria that reflect ES values beyond those related to species conservation is a sought after goal for research on methods proposed for the design of protected areas. Research has shown that the success of protected areas is closely related to socio-economic well-being of user groups and various other anthropocentric objectives [3, 22, 42, 64]. The ES approach gives expression to human needs. Despite the importance of the ES approach and much literature on the topic, leaders have been slow to incorporate the approach into decision making [31, 39, 61]. By its use of anthropocentric high-level criteria including seascape values, water sport and commercial values, this method incorporates a broad set of ES values.

In addition to allowing the incorporation human dimensions in protected area planning, an advantage of the method proposed (over other spatial conservation prioritization approaches) is that it is relatively easy to understand, to articulate and similarly easy to update as conditions change over time. Such updates and changes are as important for socio-economic related attribute data and preferences (e.g., [65]) as they are for the rapidly changing ecological conditions of the eastern Mediterranean Sea due to such factors as ocean warming and the spread of invasive species (e.g., [66]). Further stakeholder input can be incorporated, for example by administering the questionnaire to a larger number of stakeholders, and new physical attribute data as well as other high-level criteria can be applied.

Other than the comparative (Eq 2) above that considers all of the cell grades relative to other grades in the spatial plane, physical and socio-economic values are simply weighted and summed. If the MCA includes a very detailed tracking of decision points and data quality concerns, replication and adjustment of the analysis can occur over time and explanation of the process to decision makers and stakeholders should be straight forward. Further, physical attribute rankings can reflect better data (including new data points) and greater certainty over time. Since the method used in this study puts significant emphasis on the spatial layout of features, it is possible to compare MCA results to those of other decision support tools (e.g., Marxan and Zonation) using the protection of the same features as targets.

Yet there are a number of limitations to the study. These reflect mostly on the application of the method for this research and they could be corrected for future analyses. The number of stakeholders surveyed (59) is small, although it should be noted that previous MCA's, (e.g., [17, 19, 21]) determined high-level criteria preferences with even smaller numbers of respondents. Surveys were administered in late Fall at the end of the tourist season and the number of visitors/recreationists that could be approached were few. Survey administrators tried to maintain a balance so that there would not be an overwhelming number of respondents from any one group (e.g., field experts over reserve employees). Further, consent from the respondents for their participation was not easy to obtain, despite the limited number of questions (18). The survey took time to complete, mainly because of an almost full page of explanation regarding the protection scenarios. Also, the high-level criteria took some time to read and understand (see S1 Appendix). In any case, emphasis of this paper is not on the determination of preferences, but rather on the ranking of physical attributes (features) based on ES values.

It is clear that not all MPAs in the Mediterranean Sea have developed management plans, not all management plans include zoning and lastly, many management plans exist but are not well implemented or enforced [8, 67]. Yet through the use of MCA for the design of zoning within an MPA, planners have a number of different scenarios to consider that would be acceptable to user groups and likely supported. However, further research efforts are needed to render this type of MCA, so dependent on resource user valuation, capable of incorporating socio-economic values based on ES away from the shore in areas of low accessibility and low familiarity. Therefore, an aim of research taking place to improve the incorporation of the ecosystems services approach in decision making (e.g., [39]), especially with regards to the marine environment, should improve knowledge of ES values at significant distances from shore.

## Supporting Information

S1 Appendix Stakeholder questionnaire. (DOCX)

S2 Appendix The sign (sgn) function in matrix form. (DOCX)
