# Supplementary material for: Incorporation of Socio-Economic Features' Ranking in Multicriteria Analysis Based on Ecosystem Services for Marine Protected Area Planning
Source: PLoS One. 2016 May 16;11(5):e0154473. doi: 10.1371/journal.pone.0154473 (PMC4868350; doi:10.1371/journal.pone.0154473)
Supplement: S1 Appendix — (DOCX) [file pone.0154473.s001.docx]

**S1 Appendix. Stakeholder Questionnaire**

This questionnaire aims to solicit your opinion about the environmental values of the Marine Reserve Rosh Hanikra-Achziv, as part of a research project of planning marine reserves. The goal is to test the application of a model that uses a several planning techniques as well as the use of geographic information systems (GIS). Implementation of the model will result in the development of proposed zoning alternatives.

The emphasis in the study is on the examination and cataloging of the various values in the ​​proposed reserve area, and the impact of these values ​​on the planning process of the marine reserve. The emphasis is not on the detail of the maps or specific areas proposed. Resulting maps will be for academic purposes only and not for any purpose other than study and research.

The importance of different elements of the marine reserve will be determined based on questionnaire answers. The possible zoning alternatives and value types (attributes) used in this study are explained below.

Your preferences and those of other participating stakeholders will be translated into attribute rankings (weights). A set of rankings will be determined for each stakeholder group; each group has equal standing.

The questionnaires are filled out anonymously and as mentioned, the use of your answers will be only for research purposes. The questionnaire should be answered individually and there are no wrong or right answers.

**Thank you very much for participating!**

For more information about the research contact: Dr. Michelle Portman, Faculty of Architecture and Town Planning Technion – Israel Institute of Technology, [michellep@cc.technion.ac.il](mailto:michellep@cc.technion.ac.il)

PART A: Please familiarize yourself with the following definitions before answering the questions in Part B.

| **Levels of protection (scenarios)** | **Objectives** | **Operative meaning** |
| --- | --- | --- |
| **Highest level of preservation.** | To conserve the biological and genetic resources, representing the ecosystem(s) of the reserve. Management of the area is designed to protect natural resources and environmental quality. | No entry in most areas of the reserve. There is almost no entry allowed throughout the reserve |
| **Medium level conservation** | Management to achieve sustainable use of marine resources. Use will be minimal, focused and controlled. | Entry and use of the reserve is allowed in certain areas. The restrictions and prohibitions will be use-specific and area specific. No entry allowed to some specific areas of the reserve. |
| **Marine Park** | Management of the area focuses on human use while maintaining unique natural features found within the reserve. | A variety of uses are allowed. Most of the park area is open to entry and use with very few restrictions |

| **Physical attributes (“values”)** | **Definition** |
| --- | --- |
| **Marine nature values** | Physical elements/resources of the environment such as: biodiversity, species composition, water quality, etc. |
| **Marine landscape values** | Landscape characteristics of the natural environment, such as: abrasion tables, submarine canyons, inaccessible archaeological sites, etc. |
| **Cultural values and nautical sports** | Resources of active and passive recreation (water sports) conducted on an individual basis (without the support of a place-based organized business): such as: diving, surfing, canoeing, snorkeling, accessible archaeological sites, etc. |
| **Commercial values** | Areas and attributes used commercially for: fishing, boat tours, tourist attractions, organized sports, diving and surfing courses, transport and rental of boats, etc. |

PART B: Below are 6 questions for each of the three zoning alternatives – a total of 18 questions. Compare each two values ​​shown in each of the tables, one versus the other. Mark the box that expresses most accurately (closest to your views) the relative importance of values ​​for each of the alternatives (1 thru 3).

**1. Highest level of preservation**:

| 1a. | **Marine nature values** | | | **Marine landscape values** | | |
| --- | --- | --- | --- | --- | --- | --- |
| check one | __________________________________________________________________________ | | | | | |
|  |  |  |  | |  |  |
|  | much more important | slightly more important | equally important | | slightly more important | much more important |

| 1b. | **Marine nature values** | | | **Cultural values and nautical sports** | | |
| --- | --- | --- | --- | --- | --- | --- |
| check one | __________________________________________________________________________ | | | | | |
|  |  |  |  | |  |  |
|  | much more important | slightly more important | equally important | | slightly more important | much more important |

| 1c. | **Marine nature values** | | | **Marine commerce values** | | |
| --- | --- | --- | --- | --- | --- | --- |
| check one | __________________________________________________________________________ | | | | | |
|  |  |  |  | |  |  |
|  | much more important | slightly more important | equally important | | slightly more important | much more important |

| 1d. | **Marine landscape values** | | | **Cultural values and nautical sports** | | |
| --- | --- | --- | --- | --- | --- | --- |
| check one | __________________________________________________________________________ | | | | | |
|  |  |  |  | |  |  |
|  | much more important | slightly more important | equally important | | slightly more important | much more important |

| 1e. | **Marine landscape values** | | | **Marine commerce values** | | |
| --- | --- | --- | --- | --- | --- | --- |
| check one | __________________________________________________________________________ | | | | | |
|  |  |  |  | |  |  |
|  | much more important | slightly more important | equally important | | slightly more important | much more important |

| 1f. | **Cultural values and nautical sports** | | | **Marine commerce values** | | |
| --- | --- | --- | --- | --- | --- | --- |
| check one | __________________________________________________________________________ | | | | | |
|  |  |  |  | |  |  |
|  | much more important | slightly more important | equally important | | slightly more important | much more important |

**2. Medium level conservation**

| 2a. | **Marine nature values** | | | **Marine landscape values** | | |
| --- | --- | --- | --- | --- | --- | --- |
| check one | __________________________________________________________________________ | | | | | |
|  |  |  |  | |  |  |
|  | much more important | slightly more important | equally important | | slightly more important | much more important |

| 2b. | **Marine nature values** | | | **Cultural values and nautical sports** | | |
| --- | --- | --- | --- | --- | --- | --- |
| check one | __________________________________________________________________________ | | | | | |
|  |  |  |  | |  |  |
|  | much more important | slightly more important | equally important | | slightly more important | much more important |

| 2c. | **Marine nature values** | | | **Marine commerce values** | | |
| --- | --- | --- | --- | --- | --- | --- |
| check one | __________________________________________________________________________ | | | | | |
|  |  |  |  | |  |  |
|  | much more important | slightly more important | equally important | | slightly more important | much more important |

| 2d. | **Marine landscape values** | | | **Cultural values and nautical sports** | | |
| --- | --- | --- | --- | --- | --- | --- |
| check one | __________________________________________________________________________ | | | | | |
|  |  |  |  | |  |  |
|  | much more important | slightly more important | equally important | | slightly more important | much more important |

| 2e. | **Marine landscape values** | | | **Marine commerce values** | | |
| --- | --- | --- | --- | --- | --- | --- |
| check one | __________________________________________________________________________ | | | | | |
|  |  |  |  | |  |  |
|  | much more important | slightly more important | equally important | | slightly more important | much more important |

| 2f. | **Cultural values and nautical sports** | | | **Marine commerce values** | | |
| --- | --- | --- | --- | --- | --- | --- |
| check one | __________________________________________________________________________ | | | | | |
|  |  |  |  | |  |  |
|  | much more important | slightly more important | equally important | | slightly more important | much more important |

**3. Marine Park**

| 3a. | **Marine nature values** | | | **Marine landscape values** | | |
| --- | --- | --- | --- | --- | --- | --- |
| check one | __________________________________________________________________________ | | | | | |
|  |  |  |  | |  |  |
|  | much more important | slightly more important | equally important | | slightly more important | much more important |

| 3b. | **Marine nature values** | | | **Cultural values and nautical sports** | | |
| --- | --- | --- | --- | --- | --- | --- |
| check one | __________________________________________________________________________ | | | | | |
|  |  |  |  | |  |  |
|  | much more important | slightly more important | equally important | | slightly more important | much more important |

| 3c. | **Marine nature values** | | | **Marine commerce values** | | |
| --- | --- | --- | --- | --- | --- | --- |
| check one | __________________________________________________________________________ | | | | | |
|  |  |  |  | |  |  |
|  | much more important | slightly more important | equally important | | slightly more important | much more important |

| 3d. | **Marine landscape values** | | | **Cultural values and nautical sports** | | |
| --- | --- | --- | --- | --- | --- | --- |
| check one | __________________________________________________________________________ | | | | | |
|  |  |  |  | |  |  |
|  | much more important | slightly more important | equally important | | slightly more important | much more important |

| 3e. | **Marine landscape values** | | | **Marine commerce values** | | |
| --- | --- | --- | --- | --- | --- | --- |
| check one | __________________________________________________________________________ | | | | | |
|  |  |  |  | |  |  |
|  | much more important | slightly more important | equally important | | slightly more important | much more important |

| 3f. | **Cultural values and nautical sports** | | | **Marine commerce values** | | |
| --- | --- | --- | --- | --- | --- | --- |
| check one | __________________________________________________________________________ | | | | | |
|  |  |  |  | |  |  |
|  | much more important | slightly more important | equally important | | slightly more important | much more important |

***Thank you for your participation!***
