# Supplementary material for: Incorporation of Socio-Economic Features' Ranking in Multicriteria Analysis Based on Ecosystem Services for Marine Protected Area Planning
Source: PLoS One. 2016 May 16;11(5):e0154473. doi: 10.1371/journal.pone.0154473 (PMC4868350; doi:10.1371/journal.pone.0154473)
Supplement: S2 Appendix — (DOCX) [file pone.0154473.s002.docx]

**S2 Appendix. The sgn function in matrix form.**

*e_ji_* is the value of variable *j* for observation *i*

|  | *e _j1_* | *e_j2_* | *e_j3_* | *e_j4_* | *e_j5_* | *e_j6_* | *e_j7_* | Σ |
| --- | --- | --- | --- | --- | --- | --- | --- | --- |
| *e_j1_* | 0 | *sgn(e_j1_* - *e_j2_)* | *sgn(e_j1_* - *e_j3_)* | …. | …. |  |  |  |
| *e_j2_* | *sgn(e_j2_* - *e_j1_)* | 0 |  | …. |  |  |  |  |
| *e_j3_* | *sgn(e_j3_* - *e_j1_)* | *sgn(e_j3_* - *e_j2_)* | 0 |  |  |  |  |  |
| *e_j4_* | .  .  . |  |  | 0 |  |  |  |  |
| *e_j5_* |  |  |  |  | 0 |  |  |  |
| *e_j6_* |  |  |  |  |  | 0 |  |  |
| *e_j7_* |  |  |  |  |  |  | 0 |  |
